# Immunomodulatory and Antioxidant Effects of Polysaccharides from the Parasitic Fungus *Cordyceps kyushuensis*

**DOI:** 10.1155/2020/8257847

**Published:** 2020-08-29

**Authors:** Jinjuan Su, Jing Sun, Tongtong Jian, Guoying Zhang, Jianya Ling

**Affiliations:** ^1^State Key Laboratory of Microbial Technology, Shandong University, Qingdao, Shandong 266237, China; ^2^Dezhou People's Hospital, Dezhou, Shandong 253056, China; ^3^Shandong University of Traditional Chinese Medicine, Jinan Shandong 250014, China

## Abstract

The ascomycete Cordyceps genus has been used as valued traditional Chinese medicine. *Cordyceps kyushuensis* is a unique species of Cordyceps, which parasitizes on the larvae of *Clanis bilineata* Walker, and its major component cordycepin and aqueous extract are known to have many pharmacological effects. However, the physiological function of water-soluble polysaccharides has not been explored in detail. In this study, to resolve these doubts, we extracted and separated Cordyceps-derived polysaccharides and then evaluated the immunomodulatory and antioxidant activities. Four polysaccharide fractions were purified from Cordyceps-cultured stroma by DEAE-cellulose 23 and Sephadex G-150 column chromatography. Basic structural information was elucidated on the basis of physicochemical property and spectroscopic evidences. The antioxidant activities were evaluated by a 1,1-diphenyl-2-picrylhydrazyl (DPPH) radical method and protective effect of DNA damage. The qualified immunologic activities were also determined *in vivo* and *in vitro*. The polysaccharides could stimulate the proliferation of mouse splenocytes whether concanavalin A (ConA) and lipopolysaccharide (LPS) existed or not, strengthen peritoneal macrophages to devour neutral red, and increase the content of interleukin-2 (IL-2) and tumor necrosis factor-alpha (TNF-*α*) in serum. The research provides the corresponding evidence for Cordyceps polysaccharides as a potential candidate for functional foods and therapeutic agents.

## 1. Introduction

It is reported that about a total of 110,000 fungal species had been recognized [1]. Macrofungi, also known as mushrooms, have been extensively used as foods, nutraceuticals, and medicines since time immemorial [2]. The edible and medicinal mushrooms are recognized as one of the most important food supplements and have been recently studied for bioactive metabolites because of their vital roles in human health, nutrition, and various illnesses. Fungal-derived polysaccharides, a kind of natural biological macromolecules, are recognized as “biological response modifiers,” mainly derived from the mycelium, fruiting bodies, and fermentation broth of the Basidiomycetes family and some from the ascomycetes. Depending on their chemical composition, molecular weight, conformation, glycosidic bond, degree of branching, etc. [3], fungal polysaccharides presented good nutritional values and pharmaceutical properties, such as immunomodulatory [4–6], antitumor [7–9], antioxidant [10], hypocholesterolemic [11, 12], hepatoprotective [13], and anti-inflammatory activities [14].

The genus Cordyceps belongs to the entomopathogenic fungi, Clavicipitaceae, and Ascomycotina [15]. *Cordyceps sinensis* and *Cordyceps militaris* have been used as traditional Chinese medicines for the effectiveness in improving lung and kidney functions, restoring health after prolonged sickness, and enhancing physical performance [16, 17]. Continuous attentions had been paid to Cordyceps-derived polysaccharides [18–21]. Polysaccharides from the stroma and mycelia culture of Cordyceps fungus have extensive health effects and pharmacological activities, such as stimulating the innate and adaptive immune responses and activating macrophage production [22, 23]. *Cordyceps kyushuensis* is a unique species of Cordyceps, and it is worthy of further investigation. As far as we know, the only host of *C. kyushuensis* is the larvae of *Clanis bilineata* Walker [24]. Two polysaccharides purified from the cultured stroma of *C. kyushuensis* were reported previously by our group, showing remarkably antioxidant effects by assays of various antioxidant *in vitro* systems [25].

Up to now, there are only a few research papers on purification of polysaccharides from *C. kyushuensis* and their immunostimulatory activity. Therefore, in the present study, four other water-soluble polysaccharides of *C. kyushuensis* were extracted, purified, and preliminarily characterized. Moreover, immune regulation and antioxidant properties of the fractions were also evaluated *in vitro* and *in vivo*. The research would serve as a good foundation for further investigation, development, and industrial application of Cordyceps-derived polysaccharides in functional food and therapeutic agents.

## 2. Materials and Methods

### 2.1. Materials and Reagents

#### 2.1.1. Biological Materials

The anamorph strain JY1A of *C. kyushuensis*, originally isolated from fresh natural specimen, was confirmed by means of both morphological and molecular methods and conserved by our lab. Cultured *C. kyushuensis* grew on solid rice medium and was obtained after about 90 days. The stroma was oven-dried at 60°C to constant weight, homogenized with a microplant crusher, and screened with an 80-mesh sieve for further experiment.

#### 2.1.2. Reagents

DEAE-cellulose 23 and Sephadex G-150 were purchased from Amersham Biosciences (Uppsala, Sweden). The standard monosaccharide (D-mannose, D-galactose, D-arabinose, D-fructose, L-rhamnose, D-glucuronic acid, D-glucosamine, and D-galactosamine), trifluoroacetic acid (TFA), 1-phenyl-3-methyl-5-pyrazolone (PMP), LPS, and ConA were obtained from Sigma-Aldrich (St. Louis, MO, USA). The RPMI 1640 medium and fetal bovine serum was provided by Gibco (Vienna, NY, USA). All other chemicals and solvents used were of analytical grade and obtained from Sinopharm (Shanghai, China).

### 2.2. Purification of Polysaccharides

300 g dry stroma powder was extracted with 3 L distilled water at 90°C for 2 hours, and the supernatant was centrifuged. The above operation was repeated three times, and the supernatant was combined. The mixtures were concentrated to one-third volume and precipitated by adding four volumes of 95% ethanol (*v*/*v*) and kept at 4°C overnight. The precipitate was collected and washed with 95% ethanol, acetone, and ethyl ether, respectively. The resulting fraction was dialyzed in cellulose membrane tubing (exclusion limit 3500 Da) against deionized water and lyophilized. Protein was removed by the Sevag method [26]. Crude polysaccharide was preliminarily separated by subfractionation with gradient final concentrations of ethanol (15%, 30%, 50%, and 95%). The fraction at the final concentration of 50% ethanol, named as *Cordyceps kyushuensis* polysaccharide (CKPS), was obtained by lyophilization and selected for further study. CKPS powder (100 mg) was dissolved in 2 mL water, and the supernatant was applied to a DEAE-23 column (50 × 2.0 cm i.d.), which was eluted with water and followed by a 4-step gradient of 0-0.32 M NaCl (0.06, 0.1, 0.16, and 0.32 M). Guided by the phenol-sulfuric acid method, the NaCl-eluted fraction with high content of sugar was collected, dialyzed, lyophilized, and purified with a Sephadex G-150 column (80 × 2.0 cm i.d.).

### 2.3. Analysis of Physicochemical Properties

Total sugar content was determined by a phenol-sulfuric acid method [27] with glucose as the standard. Protein concentration was measured with a Bradford protein assay kit (Beyotime, Shanghai, China). Sulfate content was evaluated using the barium chloride-gelatin method [28], and the content of uronic acid was assessed by the method of m-hydroxydiphenyl using galacturonic acid as the standard [29].

Identification and quantification of monosaccharide were carried out by the HPLC method [10] with some modification. The polysaccharide (5 mg) was hydrolyzed with 2 M TFA at 120°C for 4 hours in a sealed tube fulfilled with N_2_. Then, excessive acid was removed with methanol. The dried sample was dissolved in solution containing 0.3 M aqueous NaOH (0.5 mL) and 0.5 M methanol solution of PMP (0.5 mL) and incubated at 70°C for 100 min and then neutralized with 0.5 mL of HCl (0.3 M). The resulting solution was extracted with chloroform for three times. The aqueous layer was filtered through a 0.22 *μ*m nylon membrane (MSI, Westborough, MA, USA) and injected into a Kromasil 100-C_18_ column (250 × 4.6 mm i.d., 5 *μ*m) at 260 nm and at a column temperature of 25°C. The mobile phase, a solution of 0.02 M phosphate buffer (pH 6.7) : acetonitrile =80 : 20 (*v*/*v*), was eluted at a flow rate of 0.9 mL/min. Identification of the target compounds was based on the comparison with reference sugars. Calculation of the molar ratio of the monosaccharide was carried out on the basis of the peak area of the monosaccharide.

Homogeneity and absolute measure of molecular weight of the purified fractions were obtained by high-performance size exclusion chromatography (HPSEC) (Agilent Technologies, 1200 series LC system, St. Clara, CA, USA) coupled with multiangle laser light scattering (MALLS) (Wyatt Technology DAWN HELEOS II, St. Barbara, CA, USA) at 690 nm. The sample was dissolved in the mobile phase (5 mg/mL) and filtered through a 0.22 *μ*m MSI nylon syringe filter before injection. A serial column which combined TSK-Gel G-6000 PWXL (300 × 7.8 mm i.d., Tokyo, Japan) with TSK-Gel G-5000 PWXL columns was then employed to separate the samples at 30°C. The predegassed 0.2 M NaCl aqueous solution was applied as the elution buffer at a flow rate of 0.6 mL/min. Three injection operations of the polysaccharide were performed, and molecular mass values were determined by averaging these results.

FTIR spectra were recorded in the region 4000-400 cm^−1^ on a Thermo Nicolet 20sx spectrometer at 4 cm^−1^ resolution. The samples were blended with KBr powder, grounded, and pressed into 1 mm pellets. The ^1^H and ^13^C NMR spectra were recorded on a Brucker AM-400 MHz NMR spectrometer (Rheinstetten, Germany) at 25°C. The polysaccharide sample (25 mg) was exchanged 3 times with DMSO-*d*6 upon freeze-drying, redissolved in 0.5 mL DMSO-*d*6, and centrifuged prior to analysis.

### 2.4. Vertebrate Animal Study Methods

#### 2.4.1. Animal Care, Feeding, Housing, and Grouping

A total of 60 male healthy Kunming mice (8 weeks old, 20.0 ± 2.0 g), specific pathogen free (SPF) grade, were purchased from the Laboratory Animal Center of Shandong University. The mice were housed on a 12 h dark/12 h light cycle at 22 ± 1°C and 50-60% relative humidity, free to access to a standard laboratory rodent diet and water during the experiments. All procedures involving an animal study were approved by the Ethics Committee of School of Life Science of Shandong University. After being adapted to the environment for one week, the mice were randomly divided into test and control groups (10 for each).

#### 2.4.2. Assay of Immunity Activity *In Vivo*

The polysaccharide samples were dissolved in physiological saline and given intraperitoneally to mice at doses of 10 mg/kg/d for 7 consecutive days. The control group was treated with 0.2 mL physiological saline instead of the polysaccharide solution. The mice were sacrificed via cervical vertebra dislocation 24 h after the last administration. The spleen was removed aseptically and then was placed in aseptic PBS buffer. Spleen cells were harvested by gently mincing and grinding the spleen fragment through sterilized meshes (200 meshes) and centrifuged at 3000 rpm/min at 4°C for 5 min. After red blood cells were removed with erythrocyte lysis buffer, the remaining cells were washed twice and suspended to 1 × 10^6^ cells/mL by RPMI 1640 complete medium containing 10% fetal bovine serum. The spleen cells (100 *μ*L/well) were placed in a 96-well plate with a total volume of 200 *μ*L per well, in the presence of mitogen (5.0 *μ*g/mL ConA or 10.0 *μ*g/mL LPS, final concentration) or RPMI 1640 medium, and incubated at 37°C in a humidified 5% CO_2_ incubator for 48 h. Cell proliferation was determined by the CCK-8 assay (Dojindo, Kumamoto, Japan).

The blood samples were obtained from the eye orbital sinus under light ether anaesthesia prior to being sacrificed. After centrifugation at 2000 × g for 10 min, the serum samples were collected. The IL-2 and TNF-*α* concentrations were measured with a mouse IL-2 or TNF-*α* Sunny ELISA kit (MultiSciences, Hangzhou, China) according to the indication of the manufacturer.

#### 2.4.3. Assay of Immunity Activity *In Vitro*

8-12-week old SPF mice were sacrificed by cervical dislocation, spleens were collected under aseptic conditions in RPMI 1640, and spleen cells were prepared and adjusted to 1 × 10^6^ cells/mL. 100 *μ*L/well of splenocyte suspension was seeded into a 96-well culture plate and mixed with 100 *μ*L polysaccharide solutions (62.5, 125, 250, and 500 *μ*g/mL, final concentration, respectively) in triplicate. The RPMI 1640 medium was added as a blank control, and ConA (5.0 *μ*g/mL, final concentration) and LPS (10.0 *μ*g/mL, final concentration) was used as positive controls, respectively. The plate was incubated at 37°C in a humidified 5% CO_2_ incubator for 48 h. The viable cells were determined at 450 nm. The cell proliferation rate (%) was calculated as the absorbance of sample-treated cells divided by the absorbance of control cells. Cell viability of the control group was 100%.

The resident macrophages of mice were harvested by peritoneal lavage, and the cells were subsequently cultured in RPMI 1640 complete medium and diluted to a density of 2 × 10^6^ cells/mL. The purity of macrophages was tested by adherence. Macrophage suspension (100 *μ*L/well) was pipetted into a 96-well culture plate and incubated for 3 h (37°C, 5% CO_2_). The adherent macrophages were washed twice with complete medium and then incubated with 100 *μ*L various concentrations (125, 250, 500, and 1000 *μ*g/mL) of polysaccharides for 24 h. The stimulated cells were washed twice with PBS, and 100 *μ*L neutral red (0.1%, *w*/*v*) was used to assess the phagocytosis. The plate was incubated for 3 h. After the removal of unphagocytized neutral red with PBS, 200 *μ*L cell lysate (the volume ratio of acetic acid to ethanol was 1 : 1) was added in and kept for 3 h. The OD value of each well was read at 540 nm using the Model 550 Microplate Reader (Bio-Rad, Hercules, CA, USA). DMEM and LPS (10 *μ*g/mL) were used as the blank and positive controls, respectively.

#### 2.4.4. Dislocation Euthanasia Method

At the end of the experiments, the cervical vertebrae of all surviving animals were dislocated by external force and the spinal cord was severed to make them die painlessly.

### 2.5. DPPH Radical Scavenging Assay

The radical scavenging effects of the polysaccharides were estimated by using the DPPH free radical method [30]. DPPH solution (50 *μ*L, 0.1 mM) in 50% ethanol was added in a 96-well plate with equivalent aliquot sample solution at different final concentrations (0.5, 1, 1.5, 2, and 2.5 mg/mL). The reaction solution was shaken vigorously and incubated at room temperature for 30 min, and the absorbance at 517 nm was measured. Vitamin C (VC) was used as a positive control. The DPPH scavenging rate (*R*) was calculated as the following formula: *R* (%) = [1 − (*A*_s_ − *A*_i_)/A0] × 100, as indicated by the absorbance of sample or VC. Sample reference solution, which contained equivalent 50% ethanol instead of the DPPH solution, was recorded as *A*_i_, while distilled water instead of sample was used for the blank *A*_0_. All tests were performed in triplicate, and the mean of Abs was used in the equation above.

### 2.6. Determination of DNA Damage Protective Effect

DNA damage protection activities of polysaccharides were determined with pUC19 plasmid DNA, isolated from *Escherichia coli* DH5*α* with a SanPrep Column Plasmid Mini-Preps Kit (Sangon Biotech, Shanghai, China). pUC19 plasmid was damaged by H_2_O_2_ and UV treatment using the method of Yang et al. [31]. Rutin was used as a positive control. Different structural or conformational forms of plasmid DNA were separated by electrophoresis. The reaction mixture (10 mL) contained 3 mL of plasmid DNA, 5 mL of 5 mg/mL polysaccharide or 0.4 mg/mL rutin, 1 mL of 10 mmol/mL H_2_O_2_, and 1 mL of water. The mixtures were located in a super clean bench with an ultraviolet lamp (20 W). After UV irradiation lasted for 5 min at room temperature, reaction samples along with a 10× gel loading dye were analyzed on a 1% agarose gel in TBE buffer at pH 8.0 for 30 min (100 V).

### 2.7. Statistical Analysis

For each polysaccharide, three samples were prepared for the determinations of physicochemical properties and assays of every antioxidant attribute. The samples were prepared at least in triplicate to ensure reproducibility. All bioassay results were expressed as means ± standard deviation (SD). The experimental data were fitted by using the Statistical Package of the Social Sciences (SPSS) version 11.0 (SPSS Inc., Chicago, IL, USA) and subjected to an analysis of variance (ANOVA) for a completely random design. A probability of *p* < 0.05 and *p* < 0.01 was considered significant.

## 3. Results

### 3.1. Isolation and Purification

**Figure 1:**
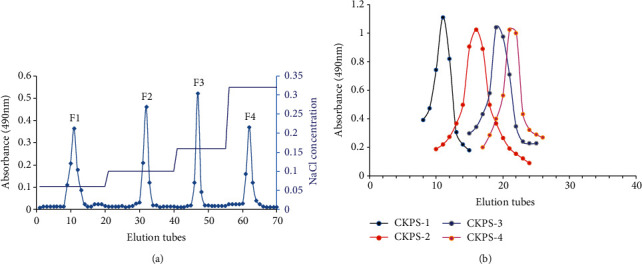
Separation and purification of polysaccharides of *Cordyceps kyushuensis*: (a) DE-23 chromatographic profile for CKPS eluted with different NaCl solutions; (b) Sephadex G-150 chromatographic profile for CKPS eluted with water.

The crude polysaccharide was obtained by water extraction, ethanol precipitation, deproteinization, and lyophilization. As shown in Figure 1(a), the 50% ethanol portion (CKPS) with a yield of 4.86% was further fractionated on a DE-23 column eluted with deionized water and at different concentrations of stepwise NaCl solution (0.06, 0.10, 0.16, and 0.32 M). Guided by the phenol-sulfuric acid method, the NaCl elutes F1, F2, F3, and F4 were further purified with a Sephadex G-150 column, respectively. Four resulting fractions shown in Figure 1(b) were named as CKPS-1, CKPS-2, CKPS-3, and CKPS-4, collected and treated for follow-up research.

### 3.2. Physicochemical Characterization

Positive response to the Bradford method and the adsorption detected by UV spectrum at 280 nm indicated the presence of protein. The results proved that CKPS-1, CKPS-2, CKPS-3, and CKPS-4 all contained minor amounts of protein (0.45, 1.07, 1.53, and 4.34%, respectively) and uronic acid (0.51, 0.77, 0.86, and 1.22%, respectively) and did not have any sulfate ester. Uronic acid was found in all four fractions, which suggested that these fractions were starch-like polysaccharides. The total carbohydrate contents of the samples were 84.35, 77.33, 84.29, and 78.22%, respectively. Neutral monosaccharide constitutions of the polysaccharides were analyzed by reversed-phase HPLC. CKPS-1 was mainly composed of Fru, Man, Glu, and Gal with molar ratios of 1 : 0.92 : 1.09 : 0.72. CKPS-2, CKPS-3, and CKPS-4 consisted of Fru, Man, and Gal in a molar percentage of 1 : 0.63 : 0.61, 1 : 1.65 : 1.4, and 1 : 2.06 : 1.97, respectively. Results showed that fructose, mannose, and galactose were the main monosaccharide components in four samples with different molar ratios and glucose was only found in CKPS-1, strongly indicating that the polysaccharides were heterogeneous. Large amounts of fructose components were found in both 50% ethanol and 90% ethanol precipitates of *C. kyushuensis* [24], which is quite different from the reports on polysaccharide of other Cordyceps species. The high-performance size exclusion chromatography (HPSEC) equipped with MALLS was considered to be a powerful, effective, and reliable technique for determining molecular characteristics of macromolecules without any calibration standard. Single and symmetrical peaks indicated that the four fractions were homogeneous polysaccharides. The weight-average molecular weight (Mw) of the purified polysaccharides was estimated to be 7153, 5945, 5643, and 5642 kDa, respectively. The IR spectra of four fractions exhibited the characteristic absorption of polysaccharides. All the fractions had similar infrared absorption bands indicating similarities in their structural features. The strong and broad peak between 3600 cm^−1^ and 3200 cm^−1^ was due to the stretching vibration of O-H. The bands at 2924 and 2854 cm^–1^, which corresponded to C-H stretching vibration in -CH_2_ and -CH_3_ groups (usually present in hexoses, like glucose or galactose, or deoxyhexoses like rhamnose or fucose), are further proven that what we are dealing with is polysaccharide containing glucuronic acid [32]. The band at 1645 cm^−1^ corresponds to the stretching vibration of the carbonyl bond that is a part of the amide group, and the band at 1545 cm^−1^ is related to the N-H bending vibration of the same group. Occurrence of these two vibrations due to the amide group indicates the presence of protein. The signal at 1408 cm^−1^ could be assigned to stretch vibration of C-O within COOH [33]. The signal at 1225 cm^−1^ accounted for asymmetric stretching vibration of the sulfate group [34]. The absorptions in the range of 1000-1200 cm^−1^, attributed to the stretching vibrations of C-O-C and C-O-H, were observed. It indicated the strong absorptions at around 1048 cm^−1^ due to stretching vibration of the pyranose ring. In addition, the absorption band at 811 cm^−1^ and 880 cm^−1^ indicated the presence of *d*-mannopyranose and galactose units [35].

**Figure 2:**
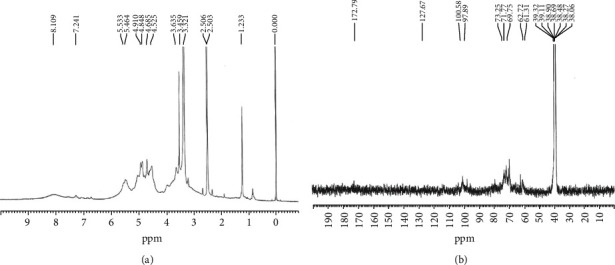
(a) ^1^H NMR spectra and (b) ^13^C NMR spectra of the purified CKPS-1 of *Cordyceps kyushuensis*.

The anomeric protons from each monosaccharide can give recognizable signals depending on *α*- or *β*-configurations. Most of *α*-anomeric protons usually appear in the 5-6 ppm region in ^1^H NMR while most of the *β*-anomeric protons in the 4-5 ppm range [36]. The signals at 5.53 and 5.46 ppm of Figure 2(a) were attributed to *α*-configuration pyranose units of CKPS-1. The resonance at 4.91 ppm may be attributed to glucosyl residues [37], and ^1^H signals at 4.53 ppm conformed to the *β*-form of D-galactopyranosyl residues. The chemical shifts from 3.4 to 4.2 ppm were assigned to protons of C-2-C-6 of the hexose glycosidic ring [38]. Thus, there were possibly both *α*- and *β*-type glycosidic linkages in CKPS-1. In a ^13^C spectrum, the signals derived from *α*-anomeric carbons usually appear in the 95-101 ppm region while most of the *β*-anomeric carbons will appear in the range 101-105 ppm [36]. The major resonance in the anomeric region occurs at 97-101 ppm rather than at 90 ppm as shown in Figure 2(b), indicating that C-1 of the *α*-monosaccharide residue is linked [39]. The signal at 172.79 ppm was due to the carboxyl resonance signal of uronic acid, which was consistent with the IR results. As judged by the absence of signals within *δ* 82-88, all sugar residues were in the pyranose form. The NMR data of other three fractions were similar to those of CKPS-1 (result not shown). The detailed structural features of the four polysaccharides should be further investigated by 2D NMR, periodate oxidation, and methylation analysis.

#### 3.2.1. Assay of Immunity Activity *In Vivo*

**Figure 3:**
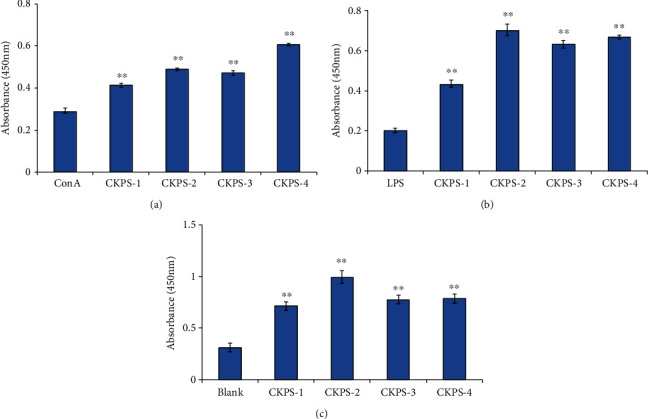
Splenocyte proliferation of polysaccharide fractions (a, b) with mitogens ConA and LPS or (c) without *in vivo*. Values are means ± SD (*n* = 3). ^∗∗^*p* < 0.01 vs. control.

In this study, Cordyceps kyushuensis is considered to be macrofungi rich in active compounds. Good immunomodulatory and antioxidant effects were evaluated in vivo and in vitro. Lymphocytes are the key effector cells of the mammalian immune system. Proliferation of splenocytes is an indicator of immune activation, being related to immunity improvement of T lymphocyte or B lymphocyte [40]. Effects of CKPS-1, CKPS-2, CKPS-3, and CKPS-4 on splenocyte proliferation with or without mitogen (ConA or LPS) are shown in Figure 3. Spleen lymphocyte proliferation induced by ConA in vivo has been used to evaluate T lymphocyte activity, while that induced by LPS has been used to examine B lymphocyte activity. The data of Figures 3(a) and 3(b) proved that, with the administration of the four polysaccharides at the doses of 10 mg/kg, the splenocyte proliferation induced by ConA or LPS was significantly enhanced (*p* < 0.01), respectively.  Figure 3(c) demonstrated that four polysaccharides still stimulated lymphocyte proliferation even without mitogenic stimuli (ConA or LPS), and the experiment results were markedly higher than those of the control medium group (*p* < 0.01). The present data also indicated that the effect of CKPS-2 on the proliferation of mixed lymphocytes (Figure 3(c)) and B lymphocytes induced by LPS (Figure 3(b)) was greater than that of the other three components, while CKPS-4 had a stronger effect on the proliferation of T lymphocytes induced by ConA (Figure 3(a)).

**Figure 4:**
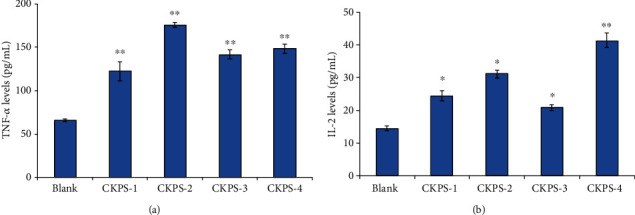
Effects of polysaccharide fractions on levels of serum (a) TNF-*α* and (b) IL-2 in mice. Values are means ± SD (*n* = 3). ^∗^*p* < 0.05, ^∗∗^*p* < 0.01 vs. control.

The IL-2 and TNF-*α* expression levels were measured to determine the stimulation properties of the immune response of the purified polysaccharide fractions. The mouse blood samples were taken from the orbit at the 24th hour after the last administration, and the serum samples were collected and ready to determine IL-2 and TNF-*α* level by extrapolation from a cytokine standard curve, according to the manufacturer's protocol. As shown in Figure 4(a), compared with the control group, the stimulating effects on the secretion of TNF-*α* were strongly enhanced by all the four fractions (*p* < 0.01). The IL-2 expression levels shown in Figure 4(b) were found to be elevated by the polysaccharides CKPS-1, CKPS-2, and CKPS-3 (*p* < 0.05). Additionally, CKPS-4 significantly promoted the secretion of IL-2 in serum (*p* < 0.01). IL-2 is essential for the growth, proliferation, and differentiation of T cells and is produced by T cells normally during an immune response [41]. TNF-*α* is a cytokine with tumor necrosis activity that is secreted mainly by macrophages and has been recognized as an important host regulatory molecule [42]. The experiment data demonstrated that the four polysaccharides could enhance the immune function by promoting cytokine expression levels for both T lymphocytes and peritoneal macrophages *in vivo*.

### 3.3. Assay of Immunity Activity *In Vitro*

**Figure 5:**
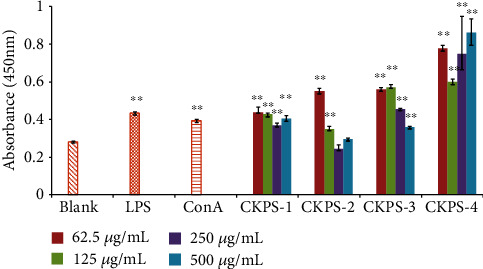
Splenocyte proliferation of polysaccharide fractions *in vitro*. Values are means ± SD (*n* = 3). ^∗∗^*p* < 0.01 vs. control.

By performing the CCK-8 assay, the effects of CKPS on normal (without mitogen) and mitogen-induced splenic lymphocyte proliferation were investigated in the final dose range of 62.5-500 *μ*g/mL. As shown in Figure 5, both ConA and LPS could greatly stimulate lymphocyte proliferation compared with the blank. Compared with the mitogen control, CKPS-4 had excellent activities on normal proliferation (*p* < 0.01). CKPS-1, CKPS-3, and CKPS-4 exhibited significant stimulation on normal proliferation at the final concentration of 62.5-500 *μ*g/mL (*p* < 0.01), but the promotion on the proliferation of lymphocytes had not shown a dose-dependent suppressive effect. At the lowest concentration of 62.5 *μ*g/mL, the proliferation rate of CKPS-2 was significantly higher than that of ConA or LPS; however, in the range of 250-500 *μ*g/mL, the high concentration of CKPS-2 did not show a stimulation effect on normal proliferation (*p* > 0.05).

**Figure 6:**
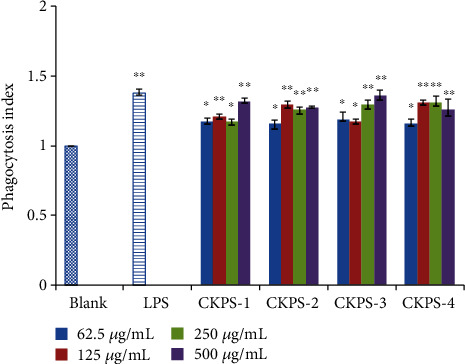
Macrophage phagocytosis of polysaccharide fractions and LPS by the neutral red uptake assay. Values are means ± SD (*n* = 3). ^∗^*p* < 0.05, ^∗∗^*p* < 0.01 vs. control.

One of the most distinguished features of activated macrophages is an increase in phagocytosis. The CKPS fractions were evaluated with regard to the effect on the phagocytic activity of macrophages using a neutral red uptake assay. As seen in Figure 6, each fraction had various enhancing effects on macrophage phagocytosis in the dose range of 62.5-500 *μ*g/mL. The phagocytic indexes of macrophages under the sample treatments all exceeded 1.0. Compared with the blank control, the fractions could considerably stimulate the phagocytosis of macrophages (*p* < 0.05 or *p* < 0.01) after administration, as well as LPS action (10 *μ*g/mL, *p* < 0.01). Macrophages played an important role in the immune system and could phagocytose aging cells, necrotic tissues, malignant cells, and pathogens invading the body and produce cytokines. The phagocytosis of macrophages was thought as one of the most important indicators of the body's nonspecific immunity [43–45]. Our present results proved that the beneficial effect of the polysaccharides on immune and inflammatory diseases might be partly attributed to the improvement of defective or deficient phagocytosis of macrophages.

### 3.4. Antioxidant Properties

**Figure 7:**
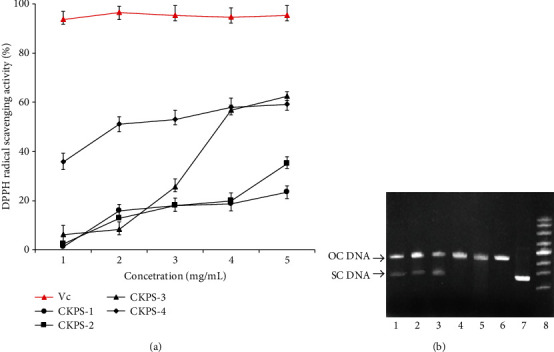
Evaluation of antioxidant activity of polysaccharides extracted from *Cordyceps kyushuensis*. (a) Scavenging activity on DPPH radical of polysaccharide fractions from *Cordyceps kyushuensis*. Values are means ± SD (*n* = 3). (b) Effects of polysaccharide fractions on the protection of supercoiled DNA (plasmid pUC19). Lanes 1-5, H_2_O_2_+UV treated with rutin, CKPS-1-4, respectively; lane 6, H_2_O_2_+UV treated without polysaccharide; lane 7, untreated DNA (control); lane 8, supercoiled DNA marker.

Natural antioxidants are known to play an important role against various diseases and aging processes. Polysaccharides were generally considered to have potential antioxidant activity. Thus, it is essential to determine the antioxidant capacities of four fractions from the stroma of *C. kyushuensis*. DPPH radical methods were often conducted to evaluate the free radical scavenging ability of natural compounds [46]. As a stable free radical, DPPH showed the maximum absorption at 517 nm with violet color due to its odd electron. When DPPH encountered antioxidant scavengers, the resulting decolorization was stoichiometric with respect to the ability to bleach the DPPH radical. The scavenging effect was measured and is shown in Figure 7(a). All the polysaccharide fractions showed a good scavenging effect against DPPH radical in a dose-dependent manner at each concentration level. The scavenging ratios at the highest concentration of CKPS-3 and CKPS-4 were 63.5% and 59.7%, respectively. And compared with other samples, especially CKPS-4 had much stronger antioxidant activity even at the low concentration of 1 mg/mL.

The protective effects of the polysaccharides on the damage induced by the coaction of H_2_O_2_ and UV were studied on pUC19 plasmid.  Figure 7(b) demonstrates the electrophoretic pattern of DNA after UV photolysis of H_2_O_2_ (2.5 mmol/L) in the absence or presence of CKPS-1 to CKPS-4 and rutin. DNA derived from pUC19 plasmid showed the band corresponding to the native form of supercoiled circular DNA (Sc DNA) on agarose gel (lane 7). After the UV irradiation of DNA with H_2_O_2_, the graph of lane 6 proved the result of the cleavage of Sc DNA to an open circular form (Oc DNA) [47]. With the addition of rutin and CKPS-1 to CKPS-4, lanes 1-5 revealed the protection effect of the polysaccharides to the damage of native Sc DNA. Lanes 2 and 3 of the gel showed clearly the Sc DNA band, which indicated that CKPS-1 and CKPS-2 had a relatively stronger capacity to suppress the formation of Oc DNA than other polysaccharides (lanes 4 and 5). The positive control of rutin (lane 1) had almost the same protective effect. A good foundation was established for the application of Cordyceps in food and pharmaceutical industry.

## 4. Discussion

Edible and medicinal mushrooms have been well known and widely consumed in far Asia as part of traditional diet and medicine for a long history for their flavor, nutrition, and biological functions. In the last decades, fungal bioactive polysaccharides have been the core of intense research for the understanding and the utilization of their special properties in naturally produced pharmaceuticals. Many biological activities of fungal polysaccharides, such as immunomodulatory, anticancer, antimicrobial, hypocholesterolemic, hypoglycemic, and health-promoting properties, have been reported. In fact, fungal bioactive polysaccharides produced by edible mushrooms make them also very good candidates for the formulation of novel functional foods and nutraceuticals without any serious safety concerns.

It has been reported that the immune response to fungal polysaccharide mixture may differ from that of purified ones [48], and as we know, the presence of other compounds, such as proteins, polyphenols, and lipids, can affect the biological activity of the fungal components. Purification and structural characterization of fungal polysaccharides are thus very important for their further application as selective and effective immune modulators [49]. The present study was undertaken to elucidate the antioxidant and immune stimulatory activities of the polysaccharides from the stoma of *C. kyushuensis*. Four water-soluble homogeneous polysaccharide fractions were isolated at the final ethanol concentration of 50% and purified by column chromatography. Preliminary structural characterizations were conducted, and DPPH scavenging activity and protection to DNA damage in vitro were carried out to evaluate the antioxidant potential of these fractions. The four polysaccharides could significantly enhance the splenocyte proliferation with or without mitogen (ConA or LPS) *in vivo* and *in vitro*. The effects on the production of cytokines IL-2 and TNF-*α* were investigated. The results showed that the levels of serum IL-2 and TNF-*α* were increased significantly by the fraction administration compared with those of the control group, suggesting that the physiological effect of the polysaccharides was implemented by increasing the immune response. Moreover, the tests of macrophage phagocytosis offered demonstrative evidence that these polysaccharides could effectively activate macrophage response. The results indicated that the polysaccharides of *C. kyushuensis* could be applied to the potential health and functional food source. This may provide new strategies for the discovery of effective and safe approaches for cancer treatment from natural resources. Of course, with the deepening of follow-up research, if several prerequisites such as economically feasible production of fungal polysaccharides with stable and standardized quality, composition, purity, and homogeneity, understanding the structure-activity relationship of bioactive polysaccharides, the molecular interaction between polysaccharides and other food ingredients, and the influence of food processing on their functions can be solved smoothly, there will be a bright future waiting for the industrial production of health products and functional food from Cordyceps polysaccharides.

## Data Availability

All data was provided in the article, and there are no more data to be uploaded.

## References

[1] Wasser S. P. (2010). Medicinal mushroom science: history, current status, future trends, and unsolved problems. *Internatinal Journal of Medicinal Mushrooms*.

[2] Friedman M. (2016). Mushroom polysaccharides: chemistry and antiobesity, antidiabetes, anticancer, and antibiotic properties in cells, rodents, and humans. *Food*.

[3] Methacanon P., Madla S., Kirtikara K., Prasitsil M. (2005). Structural elucidation of bioactive fungi-derived polymers. *Carbohydrate Polymers*.

[4] Fan S.-T., Nie S.-P., Huang X.-J. (2018). Protective properties of combined fungal polysaccharides from Cordyceps sinensis and Ganoderma atrum on colon immune dysfunction. *International Journal of Biological Macromolecules*.

[5] Cheung J.-K., Li J., Cheung A. W. (2009). Cordysinocan, a polysaccharide isolated from cultured Cordyceps, activates immune responses in cultured T-lymphocytes and macrophages: signaling cascade and induction of cytokines. *Journal of Ethnopharmacology*.

[6] Li R.-Y., Zhang L.-N., Tang Z.-Q. (2019). Effects of fungal polysaccharide on oxidative damage and TLR4 pathway to the central immune organs in Cadmium intoxication in chickens. *Biological Trace Element Research*.

[7] Rashid S., Unyayar A., Mazmanci M. A., McKeown S. R., Banat I. M., Worthington J. (2011). A study of anti-cancer effects of Funalia trogii in vitro and in vivo. *Food and Chemical Toxicology*.

[8] Fisher M., Yang L.-X. (2002). Anticancer effects and mechanisms of polysaccharide-K (PSK): implications of cancer immunotherapy. *Anticancer Research*.

[9] Wu F.-Y., Yan H., Ma X.-N. (2012). Comparison of the structural characterization and biological activity of acidic polysaccharides from Cordyceps militaris cultured with different media. *World Journal of Microbiology & biotechnology*.

[10] Sun H.-H., Mao W.-J., Chen Y., Guo S.-D., Li H.-Y., Qi X.-H. (2009). Isolation, chemical characteristics and antioxidant properties of the polysaccharides from marine fungus Penicillium sp. F23-2. *Carbohydrate Polymers*.

[11] Jin M., Zhang H., Wang J. (2019). Response of intestinal metabolome to polysaccharides from mycelia of Ganoderma lucidum. *International Journal of Biological Macromolecules*.

[12] Liu R.-M., Dai R., Luo Y., Xiao J.-H. (2019). Glucose-lowering and hypolipidemic activities of polysaccharides from Cordyceps taii in streptozotocin-induced diabetic mice. *BMC Complementary and Alternative Medicine*.

[13] Zhang Z.-F., Lv G.-Y., Pan H.-J., Pandey A., He W.-Q., Fan L.-F. (2012). Antioxidant and hepatoprotective potential of endo-polysaccharides from Hericium erinaceus grown on tofu whey. *International Journal of Biological Macromolecules*.

[14] Gondim D. V., Costa J. L., Rocha S. S., Brito G. A. D., Ribeiro R. D., Vale M. L. (2012). Antinociceptive and anti-inflammatory effects of electroacupuncture on experimental arthritis of the rat temporomandibular joint. *Canadian Journal of Physiology and Pharmacology*.

[15] Tang W., Eisenbrand G. (1992). *Chinese Drugs of Plant Origin: Chemistry, Pharmacology and Use in Traditional and Modern Medicine*.

[16] Xia Y.-L., Luo F.-F., Shang Y.-F., Chen P.-L., Lu Y.-Z., Wang C.-S. (2017). Fungal cordycepin biosynthesis is coupled with the production of the safeguard molecule pentostatin. *Cell Chemical Biology*.

[17] Bi S.-X., Huang W.-J., Chen S. (2020). Cordyceps militaris polysaccharide converts immunosuppressive macrophages into M1-like phenotype and activates T lymphocytes by inhibiting the PD-L1/PD-1 axis between TAMs and T lymphocytes. *International Journal of Biological Macromolecules*.

[18] Chen C., Wang M.-L., Jin C. (2015). Cordyceps militaris polysaccharide triggers apoptosis and G_0_/G_1_ cell arrest in cancer cells. *Journal of Asia-Pacific Entomology*.

[19] Wang Y., Liu D., Zhao H. (2014). Cordyceps sinensis polysaccharide CPS-2 protects human mesangial cells from PDGF-BB-induced proliferation through the PDGF/ERK and TGF-*β*_1_/Smad pathways. *Molecular and Cellular Endocrinology*.

[20] Wu J., Zhou J.-X., Lang Y.-G. (2012). A polysaccharide from Armillaria mellea exhibits strong in vitro anticancer activity via apoptosis-involved mechanisms. *International Journal of Biological Macromolecules*.

[21] Hu S., Wang J., Li F. (2019). Structural characterisation and cholesterol efflux improving capacity of the novel polysaccharides from Cordyceps militaris. *International Journal of Biological Macromolecules*.

[22] Chiu C. H., Chyau C. C., Chen C.-C., Lin C.-H., Cheng C.-H., Mong M. C. (2014). Polysaccharide extract of Cordyceps sobolifera attenuates renal injury in endotoxemic rats. *Food and Chemical Toxicology*.

[23] Meng L.-Z., Feng K., Wang L.-Y. (2014). Activation of mouse macrophages and dendritic cells induced by polysaccharides from a novel Cordyceps sinensis fungus UM01. *Journal of Functional Foods*.

[24] Shimizu D. (1994). *Color Iconography of Vegetable Wasps and Plant Worms*.

[25] Zhang G.-Y., Yin Q.-S., Han T. (2015). Purification and antioxidant effect of novel fungal polysaccharides from the stroma of Cordyceps kyushuensis. *Industrial Crops and Products*.

[26] Matthaei J. H., Jones O. W., Martin R. G., Nirenberg M. W. (1962). Characteristics and composition of RNA coding units. *Proceedings of the National Academy of Sciences of the United States of America*.

[27] Chaplin M. F., Kennedy J. F. (1994). *Carbohydrate Analysis*.

[28] Kawai Y., Seno N., Anno K. (1969). A modified method for chondrosulfatase assay. *Analytical Chemistry*.

[29] Bitter T., Muri H. M. (1962). A modified uronic acid carbazole reaction. *Analytical Chemistry*.

[30] Blois M. S. (1958). Antioxidant determinations by the use of a stable free radical. *Nature*.

[31] Yang X.-L., Wang R.-F., Zhang S.-P. (2014). Polysaccharides from Panax japonicus C.A. Meyer and their antioxidant activities. *Carbohydrate Polymers*.

[32] Leandro S. M., Gil M. C., Delgadillo I. (2003). Partial characterisation of exopolysaccharides exudated by planktonic diatoms maintained in batch cultures. *Acta Oecologica-International Journal of Ecology*.

[33] Kacurakova M., Capek P., Sasinkova V., Wellner N., Ebringerova A. (2000). FT-IR study of plant cell wall model compounds: pectic polysaccharides and hemicelluloses. *Carbohydrate Polymers*.

[34] Zhang Q.-B., Qi M., Zhao T.-T. (2005). Chemical characteristics of a polysaccharide from Porphyra capensis (Rhodophyta). *Carbohydrate Research*.

[35] Shingel K. I. (2002). Determination of structural peculiarities of dexran, pullulan and *γ*-irradiated pullulan by Fourier-transform IR spectroscopy. *Carbohydrate Research*.

[36] Cui S.-W. (2005). *Food Carbohydrates: Chemistry, Physical Properties, and Applications*.

[37] Seymour F. R. (1979). Correlation of the structure of dextrans to their 1H NMR spectra. *Carbohydrate Research*.

[38] Chauveau C., Talaga P., Wieruszeski J. M., Strecker G., Chavant L. (1996). A water-soluble *β*-d-glucan from Boletus erythropus. *Phytochemistry*.

[39] Uzochukwu S., Balog E., Loefler R. T., Ngoddy P. O. (2002). Structural analysis by ^13^C-nuclear magnetic resonance spectroscopy of glucan extracted from natural palm wine. *Food Chemistry*.

[40] Zhao H., Wang Q.-H., Sun Y.-P. (2014). Purification, characterization and immunomodulatory effects of Plantago depressa polysaccharides. *Carbohydrate Polymers*.

[41] Malek T. R. (2008). The biology of interleukin-2. *Annual Review Immunology*.

[42] Vilcek J., Lee T. H. (1991). Tumor necrosis factor. new insights into the molecular mechanisms of its multiple actions. *Journal of Biological Chemistry*.

[43] Thambiraja S. R., Phillips M., Koyyalamudi S. R., Reddy N. (2015). Antioxidant activities and characterisation of polysaccharides isolated from the seeds of Lupinus angustifolius. *Industrial Crops and Products*.

[44] Schepetkin I. A., Quinn M. T. (2006). Botanical polysaccharides: macrophage immunomodulation and therapeutic potential. *International Immunopharmacology*.

[45] Laskin D. L. (2009). Macrophages and inflammatory mediators in chemical toxicity: a battle of forces. *Chemical Research in Toxicology*.

[46] Amarowicz R., Pegg R. B., Rahimi-Moghaddam P., Barl B., Weil J. A. (2004). Free-radical scavenging capacity and antioxidant activity of selected plant species from the Canadian prairies. *Food Chemistry*.

[47] Kumar C. G., Joo H. S., Choi J. W., Koo Y. M., Chang C. S. (2004). Purification and characterization of an extracellular polysaccharide from haloalkalophilic Bacillus sp. I-450. *Enzyme and Microbial Technology*.

[48] Snarr B., Qureshi S., Sheppard D. (2017). Immune recognition of fungal polysaccharides. *Journal of Fungi*.

[49] Baeva E., Bleha R., Lavrova E. (2019). Polysaccharides from basidiocarps of cultivating mushroom Pleurotus ostreatus: isolation and structural characterization. *Molecules*.

